# Supplementary material for: Specific depletion of the motor protein KIF5B leads to deficits in dendritic transport, synaptic plasticity and memory
Source: eLife. 2020 Jan 21;9:e53456. doi: 10.7554/eLife.53456 (PMC7028368; doi:10.7554/eLife.53456)
Supplement: Supplementary file 1. [file elife-53456-supp1.docx]

| Figure | Comparison | Test | Sample size | Age | Gender | Number of independent experiments | *p*-value | post hoc tests | F, Dfn, Dfd |
| --- | --- | --- | --- | --- | --- | --- | --- | --- | --- |
| 1A | KIF5A  P1 vs. P21 | Two-way ANOVA | 3 mice for P1, P7, P14, P21, P31 and P56 respectively | P1, P7, P14, P21, P31 and P56 | male | 3 | 0.0188 | Dunnett's multiple comparisons test | 11.91, 5, 30 |
|  | KIF5A  P1 vs. P31 |  |  |  |  |  | 0.0489 | Dunnett's multiple comparisons test | 11.91, 5, 30 |
|  | KIF5A  P1 vs. P56 |  |  |  |  |  | 0.0052 | Dunnett's multiple comparisons test | 11.91, 5, 30 |
|  | KIF5B  P1 vs. P31 |  |  |  |  |  | 0.0430 | Dunnett's multiple comparisons test | 11.91, 5, 30 |
|  | KIF5B  P1 vs. P56 |  |  |  |  |  | 0.0034 | Dunnett's multiple comparisons test | 11.91, 5, 30 |
|  | KIF5C  P1 vs. P14 |  |  |  |  |  | 0.0365 | Dunnett's multiple comparisons test | 11.91, 5, 30 |
|  | KIF5C  P1 vs. P21 |  |  |  |  |  | 0.0137 | Dunnett's multiple comparisons test | 11.91, 5, 30 |
|  | KIF5C  P1 vs. P56 |  |  |  |  |  | 0.0411 | Dunnett's multiple comparisons test | 11.91, 5, 30 |
| 1C | Amplitude Control shRNA vs. KIF5A shRNA | Kruskal-Wallis test | 13, 10, 11, 10 neurons for control shRNA, KIF5A shRNA, KIF5B shRNA and KIF5C shRNA respectively |  |  | 5 | 0.0062 | Dunn's multiple comparisons test | 4.732, 3, 40 |
|  | Frequency Control shRNA vs. KIF5B shRNA |  |  |  |  |  | 0.0333 | Dunn's multiple comparisons test | 5.291, 3, 40 |
| 1D | Mushroom Control shRNA vs. KIF5B shRNA | Kruskal-Wallis test | 50 neurons for control shRNA  47 neurons for KIF5A shRNA  42 neurons for KIF5B shRNA  55 neurons for KIF5C shRNA |  |  | 3 | 0.0003 | Dunn's multiple comparisons test | 1.185, 3, 190 |
|  | Mushroom Control shRNA vs. KIF5C shRNA |  |  |  |  |  | 0.0051 | Dunn's multiple comparisons test | 1.185, 3, 190 |
|  | Filopodia Control shRNA vs. KIF5B shRNA |  |  |  |  |  | < 0.0001 | Dunn's multiple comparisons test | 1.829, 3, 190 |
|  | Thin Control shRNA vs. KIF5B shRNA |  |  |  |  |  | 0.0205 | Dunn's multiple comparisons test | 1.176, 3, 190 |
|  | Thin Control shRNA vs. KIF5C shRNA |  |  |  |  |  | 0.0132 | Dunn's multiple comparisons test | 1.176, 3, 190 |
|  | Stubby Control shRNA vs. KIF5B shRNA |  |  |  |  |  | 0.0167 | Dunn's multiple comparisons test | 2.612, 3, 190 |
| 2A | Mushroom Control shRNA vs. KIF5B shRNA | Kruskal-Wallis test | 51 neurons for control shRNA  47 neurons for KIF5B shRNA  42 neurons for KIF5B shRNA + KIF5B  45 neurons for KIF5B shRNA + KIF5A |  |  | 3 | < 0.0001 | Dunn's multiple comparisons test | 3.064, 3, 181 |
|  | Mushroom Control shRNA vs. KIF5B shRNA+KIF5A |  |  |  |  |  | < 0.0001 | Dunn's multiple comparisons test | 3.064, 3, 181 |
|  | Mushroom KIF5B shRNA vs. KIF5B shRNA+KIF5B |  |  |  |  |  | < 0.0001 | Dunn's multiple comparisons test | 3.064, 3, 181 |
|  | Mushroom KIF5B shRNA+KIF5B vs. KIF5B shRNA+KIF5A |  |  |  |  |  | 0.0004 | Dunn's multiple comparisons test | 3.064, 3, 181 |
| 2B | Mushroom KIF5B shRNA vs. Control shRNA | Kruskal-Wallis test | 45 neurons for control shRNA  41 neurons for KIF5B shRNA  46 neurons for KIF5B shRNA+ KIF5B  34 neurons for KIF5B shRNA+ KIF5C |  |  | 4 | < 0.0001 | Dunn's multiple comparisons test | 5.545, 3, 162 |
|  | Mushroom KIF5B shRNA vs. KIF5B shRNA+KIF5B |  |  |  |  |  | 0.0044 | Dunn's multiple comparisons test | 5.545, 3, 162 |
|  | Mushroom KIF5B shRNA vs. KIF5B shRNA+KIF5C |  |  |  |  |  | 0.0022 | Dunn's multiple comparisons test | 5.545, 3, 162 |
| 3D | Total Control shRNA vs KIF5B shRNA | One-way ANOVA | 17, 19 and 18 neurons transfected with control shRNA, KIF5A-shRNA, and KIF5B-shRNA, respectively |  |  | 3 | 0.0084 | Tukey's multiple comparisons test | 1.724, 2, 51 |
|  | Total KIF5A shRNA vs KIF5B shRNA | One-way ANOVA |  |  |  |  | 0.0129 | Tukey's multiple comparisons test | 1.724, 2, 51 |
|  | Stationary Control shRNA vs KIF5A shRNA | One-way ANOVA |  |  |  |  | 0.0002 | Tukey's multiple comparisons test | 0.8393, 2, 51 |
|  | Stationary Control shRNA vs KIF5B shRNA | One-way ANOVA |  |  |  |  | < 0.0001 | Tukey's multiple comparisons test | 0.8393, 2, 51 |
|  | Oscillatory KIF5A shRNA vs KIF5B shRNA | One-way ANOVA |  |  |  |  | 0.0264 | Tukey's multiple comparisons test | 0.9111, 2, 51 |
|  | Retrograde Control shRNA vs KIF5A shRNA | Kruskal-Wallis test |  |  |  |  | 0.0406 | Dunn's multiple comparisons test | 0.7126, 2, 51 |
|  | Retrograde KIF5A shRNA vs KIF5B shRNA | Kruskal-Wallis test |  |  |  |  | 0.0440 | Dunn's multiple comparisons test | 0.7126, 2, 51 |
| 3E | Camk2a mRNA granule distribution Control shRNA vs KIF5B shRNA | Two-way ANOVA | 11, 20 and 11 neurons transfected with control shRNA, KIF5A-shRNA, and KIF5B-shRNA, respectively. |  |  | 3 | 0.0104 | Tukey's multiple comparisons test | 4.73, 2, 39 |
|  | Grin2b mRNA granule density Control shRNA vs KIF5B shRNA | Kruskal-Wallis test | 13, 20 and 16 neurons transfected with control shRNA, KIF5A-shRNA, and KIF5B-shRNA, respectively. |  |  |  | 0.0353 | Dunn's multiple comparisons test | 1.587, 2, 46 |
|  | Camk2a mRNA granule density Control shRNA vs KIF5B shRNA | One-way ANOVA | 12, 22 and 13 neurons transfected with control shRNA, KIF5A-shRNA, and KIF5B-shRNA, respectively |  |  |  | 0.0075 | Tukey's multiple comparisons test | 1.344, 2, 44 |
| 4B | Mushroom KIF5B shRNA vs. KIF5B shRNA+KIF5B | Kruskal-Wallis test | 35 neurons for KIF5B shRNA  37 neurons for KIF5B shRNA+ KIF5B  36 neurons for KIF5B shRNA+KIF5A  32 neurons for KIF5B shRNA+KIF5A/B |  |  | 3 | < 0.0001 | Dunn's multiple comparisons | 5.756, 3, 136 |
|  | Mushroom KIF5B shRNA vs. KIF5B shRNA+KIF5A/B | Kruskal-Wallis test |  |  |  |  | 0.0001 | Dunn's multiple comparisons | 5.756, 3, 136 |
|  | Mushroom KIF5B shRNA+KIF5B vs. KIF5B shRNA+KIF5A | Kruskal-Wallis test |  |  |  |  | < 0.0001 | Dunn's multiple comparisons | 5.756, 3, 136 |
|  | Mushroom KIF5B shRNA+KIF5A vs. KIF5B shRNA+KIF5A/B | Kruskal-Wallis test |  |  |  |  | < 0.0001 | Dunn's multiple comparisons | 5.756, 3, 136 |
| 5E | FMRP/FLAG | Unpaired t test | 5 replicates | | | | 0.0018 | / | / |
|  | G3BP1/FLAG | Unpaired t test | 3 replicates | | | | 0.0002 | / | / |
| 5F | Mushroom Control shRNA vs. KIF5B shRNA | Kruskal-Wallis test | 37 neurons for control shRNA,  35 neurons for KIF5B shRNA,  38 neurons for KIF5B shRNA+ KIF5B WT  35 neurons for KIF5B shRNA+ KIF5B R941/956H |  |  | 3 | 0.0003 | Dunn's multiple comparisons | 1.384, 3, 141 |
|  | Mushroom Control shRNA vs. KIF5B shRNA+KIF5B R941/956H | Kruskal-Wallis test |  |  |  |  | < 0.0001 | Dunn's multiple comparisons | 1.384, 3, 141 |
|  | Mushroom KIF5B shRNA vs. KIF5B shRNA+KIF5B | Kruskal-Wallis test |  |  |  |  | 0.0020 | Dunn's multiple comparisons | 1.384, 3, 141 |
|  | Mushroom KIF5B shRNA+KIF5B vs. KIF5B shRNA+KIF5B R941/956H | Kruskal-Wallis test |  |  |  |  | < 0.0001 | Dunn's multiple comparisons | 1.384, 3, 141 |
| 5G | Frequency KIF5B shRNA vs. KIF5B shRNA+KIF5B | Kruskal-Wallis test | 13, 14, 13 neurons for KIF5B shRNA, KIF5B shRNA+ WT KIF5B and KIF5B shRNA+ DM KIF5B shRNA respectively |  |  | 5 | 0.0060 | Dunn's multiple comparisons | 5.318, 2, 37 |
| 6B | KIF5A | One-way ANOVA | n=3 | P45±1 | All male | 2 | 0.962 | / | 0.03902, 2, 6 |
|  | KIF5B  WT vs Homo | One-way ANOVA | n=6 | P45±1 | All male except 1 WT and 1 Hetero are females |  | 0.0013 | Tukey's multiple comparisons test | 9.947, 2, 15 |
|  | KIF5C | One-way ANOVA | n=3 | P45±1 | All male |  | 0.0874 | / | 3.76, 2, 6 |
| 6C | Frontal association cortex WT vs Homo | Unpaired t test | n=3 for WT, n=3 for Homo | P42±1 | 3 males 3 females | 1 | 0.0099 | / | 39.71, 2, 2 |
| 7B | Hippocampus spine density | Mann-Whitney test | 23 dendrites from 2 WT mice and 22 dendrites from 2 Homo mice | P39±2 | All males | 1 | 0.0005 | / | 1.192, 22, 21 |
| 7C | Frontal association cortex  spine density | Mann-Whitney test | 22 dendrites from 2 WT mice and 21 dendrites from 2 Homo mice | P39±2 | All males | 1 | >0.9999 | / | 1.226, 21, 20 |
| 7D | Frequency WT vs. Homo | Mann-Whitney test | n=10 | P45±3 | All males | 6 | 0.0354 | / | 3.571, 9, 9 |
|  | Amplitude WT vs. Homo | Unpaired t test |  |  |  |  | 0.0283 |  | 3.464, 9, 9 |
| 7G | Day 0-Day 2 Elimination WT vs Hetero | One-way ANOVA | n = 6, 947 dendritic spines for WT;  n = 6, 906 dendritic spines for Hetero;  n = 6, 1078 dendritic spines for Homo. | P31±1, P33±1 | WT 1 male 5 females;  Hetero 4 males 2 females;  Homo 4 males 2 females | 9 | 0.0106 | Tukey's multiple comparisons test | 9.342, 2, 15 |
|  | Day 0-Day 2 Elimination WT vs Homo | One-way ANOVA |  |  |  |  | 0.003 | Tukey's multiple comparisons test | 9.342, 2, 15 |
|  | Day 0-Day 2 Formation | Kruskal-Wallis test |  |  |  |  | 0.3387 | / | 0.4377, 2, 15 |
| 7H | Day 2-Day 7 Elimination | One-way ANOVA |  | P33±1, P38±1 |  |  | 0.7069 | / | 0.355, 2, 15 |
|  | Day 2-Day 7 Formation WT vs Homo | One-way ANOVA |  |  |  |  | <0.0001 | Tukey's multiple comparisons test | 16.86, 2, 15 |
|  | Day 2-Day 7 Formation WT vs Hetero | One-way ANOVA |  |  |  |  | 0.023 | Tukey's multiple comparisons test | 16.86, 2, 15 |
|  | Day2-Day7 Formation Hetero vs Homo | One-way ANOVA |  |  |  |  | 0.0335 | Tukey's multiple comparisons test | 16.86, 2, 15 |
| 7I | Day0-Day2 turnover  WT vs Homo | One-way ANOVA |  | P31±1, P33±1 |  |  | 0.047 | Tukey's multiple comparisons test | 3.565, 2, 15 |
|  | Day2-Day7 turnover  WT vs Homo | Kruskal-Wallis test |  | P33±1, P38±1 |  |  | 0.0024 | Dunn's multiple comparisons | 14.2, 2, 15 |
| 7J | Reformation WT vs Homo | One-way ANOVA |  | P31±1, P38±1 |  |  | 0.0137 | Tukey's multiple comparisons test | 6.161, 2, 15 |
|  | Reformation WT vs Hetero | One-way ANOVA |  |  |  |  | 0.0372 | Tukey's multiple comparisons test | 6.161, 2, 15 |
| 8A | Social memory index  WT vs Homo | One-way ANOVA | n = 10 for WT;  n = 11 for Hetero;  n=11 for Homo. | P32±1 | WT 5 males 5 females;  Hetero 6 males 5 females; Homo 7 males 4 females | 6 | 0.0395 | Tukey's multiple comparisons test | 3.631, 2, 30 |
| 8B | NOR index WT vs Homo | One-way ANOVA | n = 11 for WT;  n = 12 for Hetero;  n = 12 for Homo. | P31±1 | WT 4 males 7 females; Hetero 6 males 6 females; Homo 5 males 7 females | 10 | 0.0195 | Tukey's multiple comparisons test | 4.361, 2, 32 |
| 8C | Fear conditioning Pre-CS WT vs CS WT | Two-way ANOVA | n=12 for WT;  n = 11 for Hetero;  n=11 for Homo. | P34±1 | WT 4 males 8 females; Hetero 5 males 6 females; Homo 8 males 3 females | 6 | <0.0001 | Tukey's multiple comparisons test | 10.01, 2, 62 |
|  | Fear conditioning Pre-CS Hetero vs Pre-CS Hetero | Two-way ANOVA |  |  |  |  | 0.0051 | Tukey's multiple comparisons test | 10.01, 2, 62 |
|  | Fear conditioning Pre-CS Homo vs Pre-CS Homo | Two-way ANOVA |  |  |  |  | 0.2113 | Tukey's multiple comparisons test | 10.01, 2, 62 |
|  | Fear conditioning CS WT vs CS Homo | Two-way ANOVA |  |  |  |  | 0.0015 | Tukey's multiple comparisons test | 10.01, 2, 62 |
|  | Fear conditioning CS Hetero vs Homo | Two-way ANOVA |  |  |  |  | 0.0110 | Tukey's multiple comparisons test | 10.01, 2, 62 |
| 8D | Barnes maze primary latency | Mann-Whitney test | n = 11 for WT;  n = 13 for Homo. | P33±2 | WT 4 males 7 females; Homo 6 males 7 females | 2 | 0.0111 | / | 49.79, 13,10 |
| 8E | fEPSP amplitude | Unpaired t test | n=6, 22 slices for WT;  n=5, 18 slices for Homo. | 3-4 month | All males | 11 | 0.0002 | / | 1.287, 21, 17 |
| 8F | Input/output | Two-way ANOVA | n=3, 11 slices for WT  n=4, 21 slices for Homo | 3-4 month | WT 1 male 2 females;  Homo 2 males 2 females | 7 | 0.9583 | / | 0.1594, 4, 116 |
| 8G | Pair-pulse ratio | Two-way ANOVA | n=3, 11 slices for WT  n=4, 21 slices for Homo | 3-4 month | WT 1 male 2 females;  Homo 2 males 2 females | 7 | 0.2415 | / | 1.390, 4, 120 |
| Fig1-figure supplement 1A | KIF5A shRNA | Multiple t test not assuming same SD | n=3 |  |  | 3 | 0.0018099 |  |  |
|  | KIF5B shRNA |  |  |  |  |  | 3.756260e-005 |  |  |
|  | KIF5C shRNA |  |  |  |  |  | 0.010686 |  |  |
| Fig1-figure supplement 1B | KIF5A shRNA | Unpaired t test | 9 cells for control shRNA;  9 cells for KIF5A shRNA |  |  | 1 | 0.0267 | / | 2.415, 8, 8 |
|  | KIF5B  shRNA | Unpaired t test | 11 cells for control shRNA;  11 cells for KIF5B shRNA |  |  | 1 | 0.0411 | / | 1.132, 10, 10 |
| Fig2-figure supplement 1B | Puncta density | Unpaired t test | 33 neurons for KIF5A and 29 neurons for KIF5B; |  |  | 2 | < 0.0001 | / | 1.781, 32, 28 |
|  | Percentage of puncta +ve spine | Mann-Whitney test |  |  |  |  | 0.0003 | / | 1.484, 28, 32 |
| Fig6-figure supplement 1C. | Frontal association cortex | Multiple t test not assuming same SD, FDR, Benjamin, Krieger, yekutieli, Q=1% | n= 3 for WT;  n=3 for Homo. | P44±3 | WT 2 males 1 female; Homo all males | 1 | no significance | / | / |
| Fig6-figure supplement 1D | Somatosensory cortex | Multiple t test not assuming same SD, FDR, Benjamin, Krieger, yekutieli, Q=1% | n= 3 for WT;  n=3 for Homo. |  |  | 1 | no significance | / | / |
| Figure 6-figure supplement 2. | KIF5B | Unpaired t test | n= 3 for WT; n=3 for Homo. |  |  | 1 | 0.0164 | / | 9.188, 2, 2 |
|  | KIF17 | Unpaired t test |  |  |  |  | 0.6224 | / | 2.355, 2, 2 |
| Fig7-figure supplement 1 | Stability | Kruskal-Wallis test | n = 6, 947 dendritic spines for WT;  n = 6, 906 dendritic spines for Hetero;  n = 6, 1078 dendritic spines for Homo | P31±1, P38±1 | WT 1 male 5 females;  Hetero 4 males 2 females;  Homo 4 males 2 females | 9 | 0.4795 | / | 0.7911, 2, 15 |
| Fig8-figure supplement 1A | OFT centre | Kruskal-Wallis test | n = 11 for WT;  n = 12 for Hetero;  n=12 for Homo. | P31±1 | WT 5 males 6 females;  Hetero 6 males 6 females;  Homo 8 males 4 females | 6 | 0.0724 | / | 2.327, 2, 32 |
|  | OFT distance | One-way ANOVA |  |  |  |  | 0.5151 | / | 0.6773, 2, 32 |
| Fig8-figure supplement 1B | EPM | Kruskal-Wallis test | n =11 for WT;  n = 12 for Hetero;  n=12 for Homo. | P31±1 | WT 5 males 6 females;  Hetero 6 males 6 females;  Homo 8 males 4 females | 6 | 0.3974 | / | 1.17, 2, 32 |
| Fig8-figure supplement 1C | MBT | One-way ANOVA | n = 7 for WT;  n = 5 for Hetero;  n = 9 for Homo. | P31±1 | WT 3 males 4 females; Hetero 3 males 2 females; Homo 6 males 3 females | 4 | 0.3607 | / | 1.08, 2, 18 |
| Fig8-figure supplement 1D | Rotarod | Two-way ANOVA | n = 5 for WT;  n = 2 for Hetero;  n = 7 for Homo. | P42±2 | WT 1 male 4 females; Hetero 1 male 1 females; Homo 4 males 3 females | 3 | 0.4501 | / | 34.19, 6, 66 |
| Fig8-figure supplement 2A | SI total interaction stage 1 | One-way ANOVA | n = 10 for WT;  n = 11 for Hetero;  n=11 for Homo. | P32±1 | WT 5 males 5 females;  Hetero 6 males 5 females; Homo 7 males 4 females | 6 | 0.4650 | / | 0.7849, 2, 31 |
|  | SI sociability inanimate Hetero vs Social 1 Hetero | Two-way ANOVA |  |  |  |  | <0.0001 | Tukey's multiple comparisons test | 0.7491, 2, 62 |
|  | SI sociability inanimate Homo vs Social 1 Homo | Two-way ANOVA |  |  |  |  | <0.0001 | Tukey's multiple comparisons test | 0.7491, 2, 62 |
|  | SI sociability index WT vs Homo | One-way ANOVA |  |  |  |  | 0.0076 | Tukey's multiple comparisons test | 5.359, 2, 30 |
| Fig8-figure supplement 2B | SI total interaction stage 2 | Kruskal-Wallis test |  |  |  |  | 0.4113 | / | 0.9143, 2, 31 |
|  | SI social memory Mouse1 WT vs Mouse2 WT | Two-way ANOVA |  |  |  |  | 0.0011 | Tukey's multiple comparisons test | 0.6457, 2, 62 |
|  | SI social memory Mouse1 Hetero vs Mouse2 Hetero | Two-way ANOVA |  |  |  |  | 0.0001 | Tukey's multiple comparisons test | 0.6457, 2, 62 |
| Fig8-figure supplement 2C | FC acquisition 200-225sec WT vs Homo | Two-way ANOVA | n = 12 for WT;  n = 11 for Hetero;  n= 11 for Homo. | P34±1 | WT 4 males 8 females; Hetero 5 males 6 females; Homo 8 males 3 females | 6 | 0.0075 | Tukey's multiple comparisons test | 0.3325, 2, 31 |
|  | FC acquisition 200-225sec Hetero vs Homo | Two-way ANOVA |  |  |  |  | 0.0136 | Tukey's multiple comparisons test | 0.3325, 2, 31 |
| Supp. Fig8-figure supplement 2D | Barnes maze acquisition 1st trial | Two-way ANOVA | n = 11 for WT;  n = 13 for Homo. | P33±2 | WT 4 males 7 females; Homo 6 males 7 females | 2 | 0.0329 | Tukey's multiple comparisons test | 10.09, 1, 23 |
|  | Barnes maze primary error | Mann-Whitney test |  |  |  |  | 0.5078 | / | 2.207, 13, 10 |
